# Case report: A pregnant woman with Crohn disease who used ustekinumab to the 3rd trimester developed severe infection

**DOI:** 10.1097/MD.0000000000036253

**Published:** 2023-12-01

**Authors:** Peng Guo, Wulan Cao

**Affiliations:** a Department of Obstetrics and Gynecology, Zhongshan City People’s Hospital, Zhongshan, China.

**Keywords:** Crohn disease, infection, pregnancy, third trimester, ustekinumab

## Abstract

**Rationale:**

Crohn disease (CD) and pregnancy often impact each other, which poses challenges for women with CD to successfully give birth to a healthy baby. The latest guideline recommends that patients with active inflammatory bowel disease delay pregnancy to induce remission and optimize disease control. Research data has showed that the incidence of infection and severe infection in patients treated with ustekinumab (UST) did not increase compared to those treated with a placebo.

**Patient concerns:**

This report describes the entire process of a pregnant woman with CD who has undergone ileostomy and long-term enteral nutrition and requires biological agents to control the disease, from conception to delivery. This case was pregnant during CD period and regularly treated with UST to the third trimester, with the onset of sepsis and septic shock at 38 weeks gestation.

**Diagnoses:**

The patient was pathologically diagnosed with CD 16 years ago and admitted to our department at 38 weeks gestation.

**Interventions:**

After admission to our department, fetal heart monitoring indicated fetal distress, so we immediately terminated the pregnancy by cesarean section. After the diagnosis of septic shock, the patient was transferred to intensive care unit for active anti-infection and symptomatic supportive treatment.

**Outcomes:**

The mother only experienced an infection in the third trimester, and cured by active treatment. The newborn was delivered at full term and confirmed to be low birth weight.

**Lessons:**

Her experience suggests that although pregnant during Crohn active period, a good outcome can be achieved through positively controlling with medication and closely monitoring it. The use of UST during pregnancy appears to be safe for both the mother and fetus but may be associated with severe infections.

## 1. Introduction

Inflammatory bowel disease (IBD) encompasses Crohn disease (CD) and ulcerative colitis. CD is a nonspecific immune-related disorder that often presents with insidious onset, chronic and recurrent disease course, lifelong relapse tendency, and poor prognosis in severe cases. CD predominantly affects individuals between 20 and 30 years of age, with a slightly higher incidence in females in higher prevalence regions.^[1]^ CD can result in fertility issues for women, as active disease can lead to decreased fertility and poor pregnancy outcomes.^[2,3]^ Conversely, pregnancy can exacerbate CD and increase the risk of surgical complications.^[4]^ Lack of understanding and guidance regarding pregnancy is common among patients with CD, making correct education and multidisciplinary support crucial for achieving positive pregnancy outcomes. This article reports the complete process of a pregnant woman with recurrent CD who underwent ileostomy, long-term enteral nutrition, and the use of ustekinumab (UST) to control the disease, from pregnancy to delivery. The literature related to the pregnancy process is reviewed to assist obstetricians in understanding and standardizing the management of these special patients and achieve favorable maternal and infant outcomes.

## 2. Case description

A 33-year-old female patient was admitted to our department on May 18, 2023 due to 38 weeks of amenorrhea and 1 day of fever. The patient’s current pregnancy was conceived naturally. Prior to conception, the patient had moderately active CD and was advised by gastroenterologists to consider pregnancy during the remission phase. However, due to urgent fertility needs, the patient attempted to conceive and succeeded. After understanding the related risks, the patient still requested to continue the pregnancy. After becoming pregnant, the patient’s CD became mildly active and was treated regularly with UST every 8 weeks in the gastroenterology department. The last treatment of UST was at 31 + 2 weeks gestation. The patient had been hospitalized for more than a month in the gastroenterology department prior to admission to the obstetrics department. Regular prenatal checkups in the obstetrics department showed no significant abnormalities. At 20 weeks gestation, the patient presented with fever which was treated symptomatically after considering novel coronavirus infection. Due to CD, the patient had been receiving enteral nutrition via a nasoenteric tube for a long time and did not eat autonomously. She started consuming a liquid diet after pregnancy, and at 35 weeks gestation, fetal growth indicators were found to be slightly smaller, particularly abdominal circumference, but she did not meet the criteria for the diagnosis of fetal growth restriction. After receiving nutritional support from the nutrition department, fetal growth improved slightly. One day prior, the patient began to experience fever, which peaked at 39°C but was reduced to normal levels after taking oral acetaminophen, and no other positive symptoms were observed.

The patient was diagnosed with colonic and penetrating CD 16 years. Despite aggressive treatment with various medications, relapses persisted. In December 2014, the patient underwent laparoscopic-assisted ileostomy and rectovaginal fistula suture hanging surgery under colonoscopy due to CD activity, recurrent anal-vaginal fistula and abscess formation, with acute allergy to infliximab. The patient remained stable without medication post-surgery. In February 2016, the patient developed abdominal discomfort, with endoscopic examination confirming relapse. After receiving enteral nutrition support treatment, the symptoms improved. However, due to the need for pregnancy, maintenance therapy was not initiated. In June 2017, the patient experienced relapse of CD that progressed to a moderate active phase. Treatment with prednisone and azathioprine resulted in some improvement, but the patient ceased medication and was not followed up. The patient reported only symptoms of hematochezia but was able to maintain a normal diet and lifestyle. On February 28, 2022, regular treatment with UST was initiated, along with complete enteral nutrition. Based on the nutritional science evaluation, the patient had been diagnosed with mild secondary protein-energy malnutrition. The patient experienced one episode of conservative treatment for partial small bowel obstruction in April 2022.

**Figure 1.**
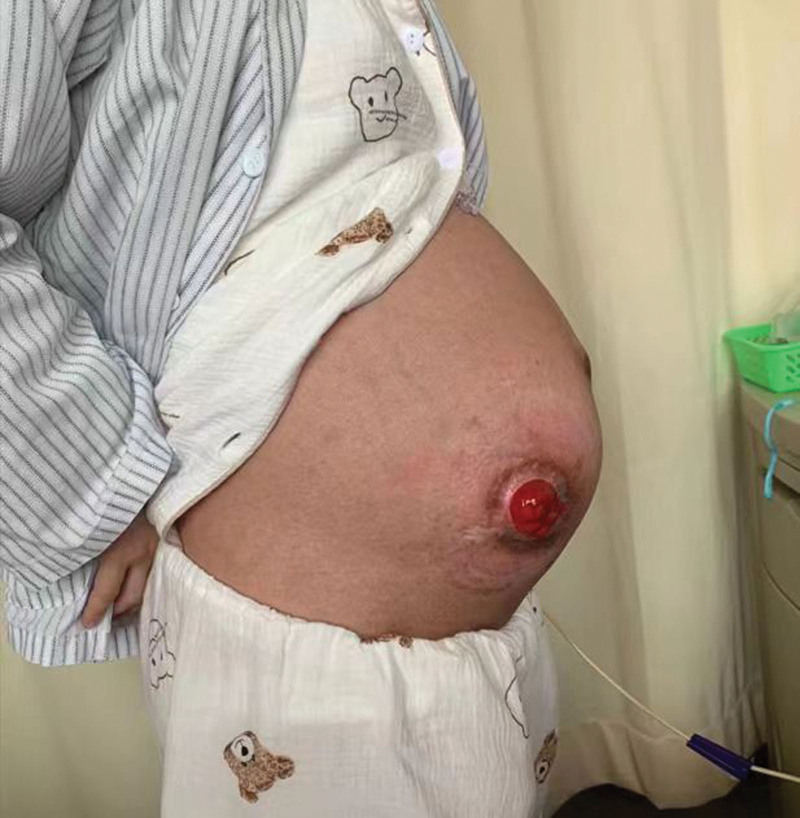
A fistula opening was observed in the lower right abdomen of the patient.

Physical examination showed the following: temperature, 37.9°C; pulse rate, 100 bpm; respiratory rate, 21 breaths/min; and blood pressure, 115/73 mm Hg. Her height is 160 cm, and her weight is 55 kg. A fistula opening was observed in her lower right abdomen (Fig. 1), but there was no tenderness or uterine contractions. Her fundal height was 31 cm and her abdominal circumference was 96 cm. The fetus was in a left occiput anterior position with a heart rate of 142 bpm. The ultrasound on May 9, 2023 showed that the fetal growth indicators were as follows: biparietal diameter was 87 mm, head circumference was 308 mm, abdominal circumference was 308 mm, and femur length was 66 mm. The placenta and amniotic fluid were normal.

**Figure 2.**
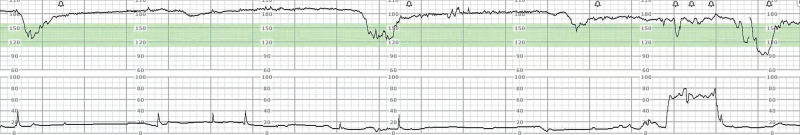
Fetal heart monitoring after admission indicated that the fetal heart rate was fast with frequent deceleration.

**Figure 3.**
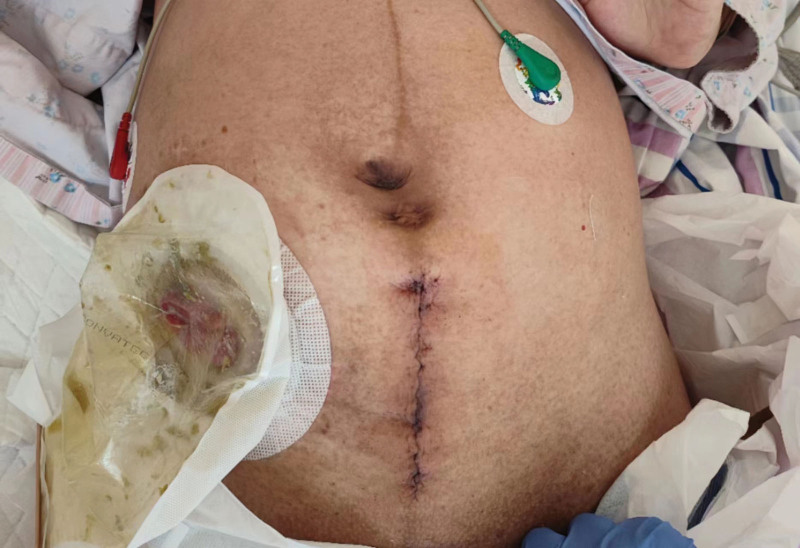
Cesarean section was performed with an abdominal median incision.

**Figure 4.**
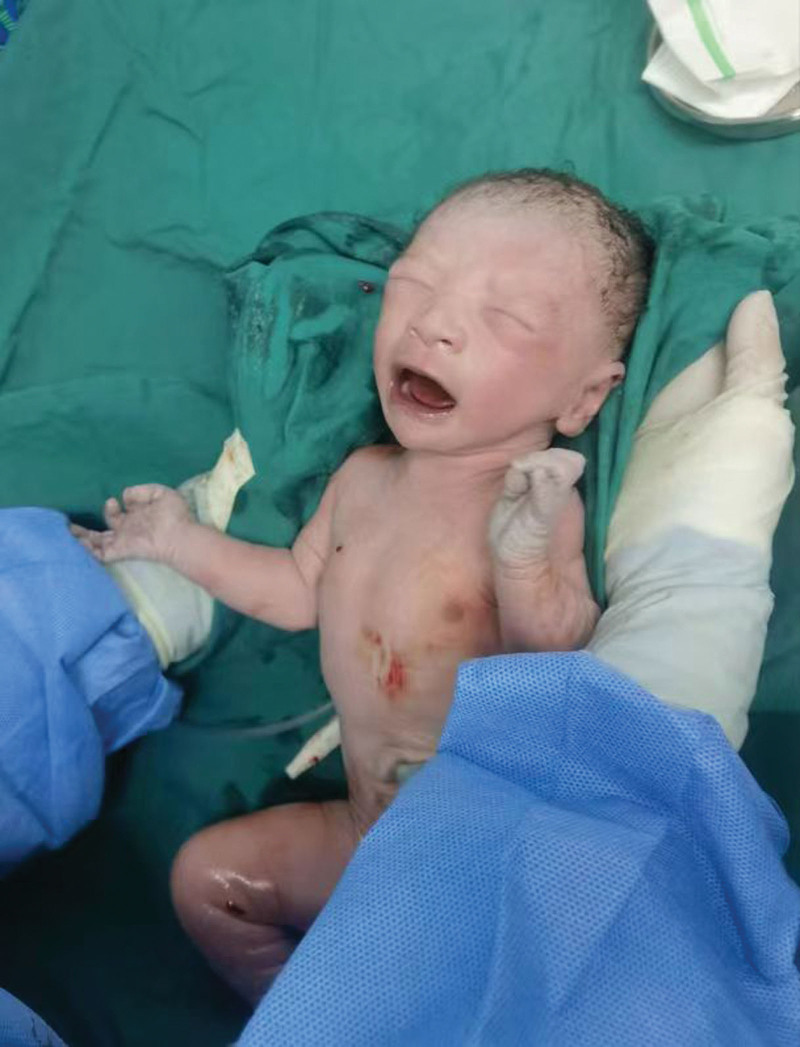
A male infant was successfully delivered.

On the day of admission to the obstetrics department, the patient’s temperature gradually rose to 39.3°C and fetal heart monitoring indicated a baseline of 180 beats per minute, with frequent decelerations suggestive of fetal distress (Fig. 2). A consultation was sought from the departments of general surgery, anesthesiology and neonatology, and an emergency cesarean section was performed with abdominal median incision (Fig. 3). The amniotic fluid was second-degree turbid. Following the procedure, a male infant weighing 2310 g and measuring 47 cm in length was successfully delivered with Apgar scores of 10-10-10 (Fig. 4). The neonate was transferred to the neonatology department for further monitoring and treatment. The patient experienced repeated high fever after surgery was diagnosed with sepsis and shock on the second day after surgery and was transferred to the intensive care unit for further treatment. The patient’s symptoms and infection indicators gradually improved with active anti-infective, anticoagulant, and parenteral nutrition support treatment. Blood culture detected *Klebsiella pneumoniae*, which was sensitive to the antibiotic meropenem. After 2 days of treatment in the intensive care unit, the patient’s condition stabilized, and she was transferred to a general ward. The patient was successfully discharged after a full course of anti-infective therapy, with blood culture showing negative results. The newborn only had elevated creatine kinase levels, but after 8 days of active treatment, the newborn was discharged smoothly. The infant was artificially fed and achieved a weight of 3300 g and a height of 52 cm at the 1-month follow-up visit after birth and was evaluated by pediatric and nutritional specialists as having good development.

## 3. Discussion

CD can affect female fertility in various ways, including ovarian dysfunction, recurrent infections, malnutrition, surgical complications such as ovarian tubal adhesion, and psychological conditions such as anxiety and depression. Research has demonstrated an increased risk of infertility in active CD patients,^[2]^ with average anti-Mullerian hormone levels in active CD patients significantly lower than those in remission CD patients and the control group.^[5]^ However, a study has shown that the fertility of patients with quiescent IBD who have no history of pelvic surgery is comparable to that of healthy individuals.^[6]^ Another study suggests that patients with severe active CD and those who have undergone surgery are at a higher risk of infertility.^[7]^ The CD in this case recurred repeatedly and was controlled by biologics for over a year. She had multiple factors of reduced fertility, such as moderate disease activity before pregnancy, previously performed ileostomy, and secondary mild malnutrition. Nonetheless, the patient’s youth, positive family environment, and optimistic mindset ultimately resulted in a natural pregnancy.

Previous research suggests that the effect of pregnancy on the activity of IBD is variable. Approximately one-third of patients experience sudden exacerbation during pregnancy, while the remaining two-thirds experience stable or improved conditions.^[8]^ The impact of pregnancy on the course of CD mainly depends on the disease activity at the onset of pregnancy and during delivery. For CD patients with stable conditions at the beginning of pregnancy, approximately 70% remain in remission throughout the entire pregnancy.^[9]^ However, for CD patients who conceive during active periods, disease exacerbation or chronic activity is often observed during pregnancy and drug treatment may be less effective. The latest Australian guidelines also recommend that patients with active IBD delay pregnancy to induce remission and optimize disease control.^[10]^ In the case of this patient, the disease was moderately active at the beginning of pregnancy but gradually stabilized to mild activity. Regular biological treatment throughout the entire pregnancy was effective, and the disease did not worsen.

IBD also affects pregnancy outcomes. Compared to non-IBD pregnant women, those diagnosed with IBD have a higher incidence of cesarean section, preterm delivery, and miscarriage, with worse outcomes observed in patients with CD compared to ulcerative colitis patients.^[11]^ IBD patients should be referred to high-risk obstetrics once pregnant and closely followed by obstetricians and gastroenterologists throughout pregnancy and the perinatal period.^[12]^ Good pregnancy outcomes for pregnant women with CD depend on the disease status during pregnancy and delivery. Studies indicate that the risk of natural abortion, pregnancy-related complications, and adverse perinatal outcomes are similar to those in the general population when the disease is quiescent during pregnancy.^[8]^ Other studies have shown that the activity of IBD is associated with miscarriage, premature birth, and low birth weight, with CD patients having a higher risk.^[13]^ Pregnant patients with active CD have an increased risk of premature birth, low birth weight, and stillbirth, with a fourfold increase in the risk of stillbirth.^[3]^ In this case, the patient’s pregnancy went full term, but in the third trimester, the fetal growth indicators gradually decreased than normal, and the newborn was ultimately confirmed as a full-term low birth weight infant.

A survey found that pregnant women with IBD are more concerned about the potential effects of medication on their fetuses than the serious consequences of active CD during pregnancy. In fact, disease activity is the greatest threat to the mother and fetus during pregnancy for women with CD, rather than drug treatment. The risks of most CD drugs during pregnancy are relatively low, and for most patients, the benefits of using medication to maintain disease stability far outweigh the adverse effects of drugs. Biologics can pass through the placenta in the third trimester. For pregnant women undergoing sustained remission, it is recommended to avoid using antitumor necrosis factor(anti-TNF) medication after 30 weeks gestation to limit fetal exposure.^[14]^ However, experts suggest continuing the use of anti-TNF medication throughout pregnancy if required to control disease.^[15,16]^ The patient in the case was treated with UST during pregnancy, which is a biologic agent that exerts biological effects by specifically binding to the common subunit of interleukin-12 (IL-12) and IL-23. Sako et al monitored pregnant women with CD who were treated with UST until 23 weeks gestation and followed the growth and development of the infants for 6 months, finally showing that UST is safe to use during pregnancy and does not increase the risk of adverse pregnancy events.^[17]^ A prospective, multicenter study on women with IBD who were continuously exposed to UST during pregnancy demonstrated favorable pregnancy and postpartum infant outcomes.^[18]^ Studies have indicated that the concentration of UST detected in breast milk is very low. Therefore, it is safe to use UST during breastfeeding, and it will not affect growth or development or lead to any complications in infants.^[19]^ The safety of using UST during pregnancy, a relatively new drug, requires more data for confirmation. This case used UST to control CD until 31 + 2 weeks gestation, and the newborn was confirmed to be a full-term low birth weight baby which might be related to disease activity during pregnancy. However, the infant showed normal growth and development without infection in the short-term follow-up after delivery. These findings suggest that the use of UST during pregnancy is safe for the fetus, and further monitoring of newborn growth and development will be conducted.

Currently, there is controversy regarding the delivery mode for pregnant women with CD, with a clinical preference for terminating pregnancy via cesarean section.^[20]^ For mild or quiescent CD during pregnancy, women should be treated as normal when determining the delivery mode, but perineotomy should be avoided. Previous studies have shown that approximately 18% of CD patients without perianal disease develop perianal lesions after vaginal delivery, particularly after perineotomy. However, numerous clinical studies have demonstrated that vaginal delivery in patients with quiescent disease does not exacerbate perianal disease.^[21]^ Cesarean section is recommended for patients who have undergone ileal pouch-anal anastomosis to reduce the risk of anal sphincter injury.^[22]^ According to European Crohn and Colitis Organisation guidelines, pregnant women with active perianal CD should avoid vaginal delivery due to the high risk of deterioration of perianal disease.^[23]^ For pregnant women with CD who had a colostomy or ileostomy, there is currently a lack of data, but it must be decided after a multidisciplinary discussion between obstetricians, colonic surgeons and gastroenterologists.^[24]^ In this paper, we report a patient with mild active CD who had undergone ileostomy and previously had rectovaginal fistula. Therefore, cesarean section was proposed as the mode of delivery before the occurrence of fetal distress. The patient was scheduled for surgery after multidisciplinary consultation, but due to sudden fever and fetal distress, emergency surgery was performed. We contacted the colorectal surgery department for assistance, but fortunately, the uterus was not adhered to the surrounding organs during the surgery, and the delivery process was successful.

The patient developed septic shock due to a concurrent infection with *Klebsiella pneumoniae* in the third trimester. *Klebsiella pneumoniae* is a common hospital-acquired pathogen and an opportunistic pathogen that can cause serious infections once entering the body. A retrospective cohort study showed that the type of IBD, disease activity, and immunosuppressive therapy do not increase the risk of perinatal infection in patients with IBD.^[25]^ Other studies have reported an increase in the incidence of infectious events accompanying the improvement of clinical efficacy in CD by biological agents.^[26]^ A meta-analysis suggests that biological agents can increase the risk of opportunistic infections in patients with IBD.^[27]^ Current data suggest that TNF-α antagonists may be associated with a 1.5 to 2 times higher risk of serious infection than other immunosuppressive agents.^[28]^ However, a comprehensive safety analysis of data from 6 phase 2/3 trials of UST showed that the incidence of infection and severe infection in patients treated with UST did not increase compared to those treated with a placebo.^[29]^ More data are needed to verify the safety of UST therapy for CD patients. The patient had been treated with UST for over a year, and although the condition was stable, she experienced a severe infection in the third trimester. It is suspected that the high-risk factors for this infection may be the activity of CD, prolonged hospitalization, and long-term use of biologics. Therefore, in addition to controlling the disease itself, treatment for CD should also take into account the risk of adverse reactions and serious infection complications associated with the use of biologics, particularly opportunistic infections.

## 4. Conclusion

Due to the overlap of the peak age of onset of CD and the reproductive age, management of pregnancy in women with CD is crucial. Pregnancy and CD have reciprocal impacts, making the management of pregnancy challenging for women with CD. Poor management may increase the risk of disease relapse and adverse pregnancy outcomes. First, it is crucial to maintain control of CD before and during pregnancy as active CD is a major predictor of adverse pregnancy outcomes. Second, current data suggest that most medications for CD, including biologics, can be safely used in pregnant women. Additionally, counseling and education for women with CD before and during pregnancy are also crucial. Many patients have unnecessary concerns and misunderstandings about fertility and pregnancy, and addressing these issues can optimize medication adherence and pregnancy outcomes. Finally, the serious infection in this case may be related to the use of UST, reminding us that the treatment of CD should not only focus on the control of the disease itself but also take into account the risk of infection after the application of biologics. Improving vigilance, early detection and early multidisciplinary cooperation can achieve good pregnancy outcomes.

## Acknowledgment

We would like to thank the patient who contributed to this study.

## Author contributions

**Formal analysis:** Peng Guo.

**Writing – original draft:** Peng Guo.

**Writing – review & editing:** Wulan Cao.
